# Will WHO Labor Care Guide have a positive effect on objectively measured health outcomes as well as patient reported measures?

**DOI:** 10.1111/aogs.14479

**Published:** 2022-12-23

**Authors:** Amar Bhide, Pål Øian, Ganesh Acharya

**Affiliations:** ^1^ Fetal Medicine Unit, Department of Obstetrics and Gynaecology St George's Hospital London UK; ^2^ Department of Obstetrics and Gynecology University Hospital of North Norway Tromsø Norway; ^3^ Women's Health and Perinatology Research Group, Department of Clinical Medicine UiT The Arctic University of Norway Tromsø Norway; ^4^ Division of Obstetrics & Gynecology, Department of Clinical Science, Intervention and Technology (CLINTEC) Karolinska Institutet and Center for Fetal Medicine, Karolinska University Hospital Stockholm Sweden

In February 2018, the World Health Organization published a set of recommendations for a positive childbirth experience.^1^ In their own words, the guideline, when delivered as a package will ensure the delivery of good‐quality and evidence‐based care irrespective of the setting or level of health care. In 2020, a user's manual was published to help health personnel to successfully use the Labor Care Guide (LCG).^2^ The usability, acceptability, and feasibility of the LCG have been tested in South America, Asia and Africa but not in high‐income settings.^3^

How did we manage to land up in this situation? Women have been giving birth since time immemorial. Are we to take it that mankind is still unsure how to look after women in childbirth or that childbirth experience is not so positive for a significant proportion of women? A summary list of all the recommendations appears at the beginning of the documents.^1^, ^2^ We will discuss the prominent ones below.

## 1 INVOLVEMENT OF WOMEN IN THEIR OWN CARE

The recommendations about providing care that is respectful and with dignity is undisputed. However, the fact that this needs to be spelt out underscores the reality that there are times when women feel that they are not respected or dealt with dignity. It is recommended that women's views be taken into account when looking after the labor process. This means different things to different people. While the accoucheurs should not be reduced to doing as they are told (by the pregnant women or her partner/relatives), they should also not be making unilateral decisions thought to be in the best interest of the woman and the baby. The extent to which women wish to make decisions about their own care is variable. Apart from personal preferences it is also driven in some parts by the culture. All clinicians have been faced with this question from the patients: ‘What would you do if you were in my situation?’ This is not an unreasonable question and difficult to respond to. The guideline authors acknowledge this diversity of expectations. Exploring the extent to which the women wish to make decisions about their care would achieve a congruity between women's expectations and their actual experience.

## 2 DIAGNOSIS OF ONSET OF LABOR

The difficulty in diagnosing onset of labor (latent as well as active phase) is well‐known to practicing obstetricians and midwives. There have been attempts by WHO to remedy this by increasing the threshold of cervical dilation from 3 cm to 4 cm in the past^4^ and to 5 cm now in LCG^1^ to diagnose active phase. Some have suggested ignoring that the latent phase exists. One would expect that the new definitions of the onset of the latent and active phase are based on outcomes such as morbidity or achievement of vaginal birth. In reality, these definitions are still based on consensus of published studies, or healthcare resource use rather than either objective health outcomes or patient reported measures. The WHO suggests avoiding using the Partograph to monitor maternal and fetal condition altogether before the onset of active phase.^1^ The idea behind it is to avoid diagnosing active phase too early and prevent interventions due to overdiagnosis of slow or lack of progress of labor. However, neither there is a consensus nor there are clear guidelines for the management of pregnant women during the period from the onset of regular painful uterine contractions until the cervix is 5 cm dilated. With the new definition, a fairly large proportion of women can be expected to be in the latent phase, who will require some monitoring and care. Therefore, clearer recommendations are desirable for the management of latent phase of labor.

## 3 ASSESSMENT OF PROGRESS OF LABOR

We have learned from studies that the ‘normal’ rate of labor progress can be variable. Definitions held until very recently may be too stringent and can result in an excess of intervention without good justification.^5^, ^6^ A slow progress of labor is often a marker of problems, although the evidence that intervention leads to improved outcomes is difficult to come by. The new recommendations acknowledge this variability in duration of the first and the second stages of labor. Rates of progress that would be considered too slow by previously held beliefs are deemed acceptable now,^7^, ^8^, ^9^ and dynamic rather than static definitions are used to diagnose slow progress. Digital vaginal examination at 4‐hourly intervals is recommended for routine assessment of active first stage of labor in low‐risk women, and alert time threshold for each cervical dilatation are set as follows: 5 cm ≥6 h, 6 cm ≥5 h, 7 cm ≥3 h, 8 cm ≥2.5 h and 9 cm ≥2 h. Although these thresholds may make sense physiologically, their interpretation may be little more complex compared to a standard 1 cm/h rule in the active phase. Furthermore, use of a new dynamic Partograph was not shown to be more effective in reducing labor dystocia or intrapartum cesarean section rate compared with the use of a traditional static WHO Partograph in a randomized controlled trial.^10^

One of the most important recommendations of the LCG is perhaps the continuing careful assessment of maternal‐fetal condition and progress of labor in the second stage. Whether this logical component, which was lacking in previous WHO Partograph, will have any effect on reducing adverse perinatal outcomes is not known and merits further investigation.

The LCG was primarily designed to be used for the care of apparently healthy pregnant women and their babies (i.e. women with low risk pregnancies), and not as a substitute for good clinical practice. Thus, it is still important to diagnose abnormal labor, such as slow progress by regular assessment throughout labor and document care plans (eg intervene or continue monitoring, setting time‐limits regarding when an intervention would be considered in consultation and agreement with the woman), as this is important both for good clinical practice and for legal reasons.

Dignity, respect and clinically as well as psychologically safe environment are equally important to the care givers and the delivering women. Continuity of care is recommended, and few would disagree. However, the practical difficulties with delivering continuity of care without compromising the personal needs of the caregivers are considerable. We are not aware of any models where both the laboring woman and the accoucheur are completely satisfied.

To state that ‘most women want a normal birth with good outcomes for mother and baby’ is a meaningless rhetoric. We cannot think of anyone who would want anything else. The contentious word is ‘good’. A debate is needed about what outcomes are important and to what extent patient‐reported outcomes and expectations should drive healthcare practices. This becomes even more complicated because judgment can get clouded in a stressful and emotionally charged situation. Can the woman really decide what is best for the situation when she is faced with extreme distress due to labor contractions? At times, health outcomes are perfect (unharmed mother and a healthy baby) but the woman is left emotionally very traumatized.

## 4 DRIVERS OF CURRENT PRACTICE

We need to acknowledge that the drivers for current practice are variable. They include healthcare setting (public/free vs private/paying), availability of resources (labor analgesia for example), differences in culture, the concern for litigation, changes in practice (higher prevalence of labor induction) and changing expectations of the society. Medical interventions during childbirth are generally intended to prevent adverse outcomes, but they may be associated with risk. Therefore, interventions without good indication are not justified. However, one cannot expect to have low intervention rate, at the same time no adverse outcome at all.

The new WHO LCG may be considered too prescriptive by some. Although, the integration of items to promote quality of care as well as positive childbirth experience for women is commendable, the caregivers' perspective is not sufficiently addressed. We would argue that the underlying principles are invariant and applicable to all settings. It is the responsibility of us health professionals to test whether the guidelines work in our specific practice environments. We look forward to the completion and subsequent publication of such a study that is at the planning stage.^11^ The effectiveness of the LCG on objectively measured health outcomes as well as patient reported measures (outcomes and experiences) should be investigated appropriately in diverse healthcare settings.
